# Head and neck lymph node region delineation with image registration

**DOI:** 10.1186/1475-925X-9-30

**Published:** 2010-06-22

**Authors:** Chia-Chi Teng, Linda G Shapiro, Ira J Kalet

**Affiliations:** 1 School of Technology, Brigham Young University, Provo, UT, USA; 2 Department of Computer Science and Engineering, University of Washington, Seattle, WA, USA; 3 Department of Radiation Oncology, University of Washington, Seattle, WA, USA

## Abstract

**Background:**

The success of radiation therapy depends critically on accurately delineating the target volume, which is the region of known or suspected disease in a patient. Methods that can compute a contour set defining a target volume on a set of patient images will contribute greatly to the success of radiation therapy and dramatically reduce the workload of radiation oncologists, who currently draw the target by hand on the images using simple computer drawing tools. The most challenging part of this process is to estimate where there is microscopic spread of disease.

**Methods:**

Given a set of reference CT images with "gold standard" lymph node regions drawn by the experts, we are proposing an image registration based method that could automatically contour the cervical lymph code levels for patients receiving radiation therapy. We are also proposing a method that could help us identify the reference models which could potentially produce the best results.

**Results:**

The computer generated lymph node regions are evaluated quantitatively and qualitatively.

**Conclusions:**

Although not conforming to clinical criteria, the results suggest the technique has promise.

## Background

Malignant tumors in the head and neck represent a great epidemiological problem in western countries. Head and neck cancer accounts for approximately 3% of all cancer cases reported in the United State, or roughly 50,000 cases per year [1]. Due to the tumor position, the risk of developing lymph node metastases in the neck region is very high. Radiation therapy is used as part of the treatment in a majority of the cases. Therefore a fast and effective system for creating a conformal radiation treatment for enlarged (i.e. potentially malignant) lymph nodes is essential.

**Figure 1:**
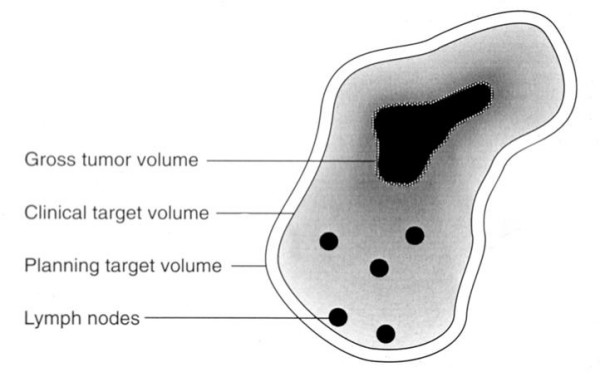
**Illustration of target volumes. (Courtesy of Mary Austin-Seymour **[25]).

Computerized tomography (CT) scanning is commonly used for conformal radiation treatment. The scan is performed with the patient set in the treatment position, immobilized using custom devices, thereby minimizing movement of the treatment target. Radiation oncologists have adopted definitions for the various components of the target volume, in order to achieve some uniformity and facilitate the conduct of inter institutional clinical trials [2,3]. The Gross Target Volume (GTV) is the visible and palpable tumor mass. Although it can usually be seen on images (CT and MR), it is normally difficult to automatically identify with existing image processing techniques. To date it is still usually hand drawn by clinicians using a computer software drawing tool. The Clinical Target Volume (CTV) includes the locations of microscopic local and regional spread, which usually means the GTV plus the lymph node regions around it. Microscopic disease cannot currently be imaged by any existing technique. Even the nodes themselves are often hard to identify in the images. The task of delineating these nodal regions, which is also usually done by the clinicians, is very time consuming. Figure 1 shows how these target volumes are related to each other [4].

Creating the 3D CTV is a critical part of the 3D radiation treatment and Intensity-Modulated Radiation Therapy (IMRT) as the success of radiotherapy depends on the accuracy of the CTV. A conformal IMRT plan with accurately drawn CTV can avoid critical anatomic structures and maximize radiation dosage. As 3D conformal radiotherapy and IMRT become the state of the art, the process of CTV delineation is more important than ever. This process currently also requires radiation oncologists to manually draw the 2D target contours on axial CT slices. It is tedious, time consuming and can be the bottle neck to make IMRT available to more patients. As imaging based cervical lymph node region classification is developed, it is possible to design a system that can identify critical anatomic structures and contour CTV by segmenting patients' CT images with little or no user interaction. Software tools that automate the segmentation of critical structures and contouring of target volume is crucial to the success of implementing a fast and effective radiation treatment planning system as it can dramatically decrease the planning effort for radiation oncologists and increase the availability of IMRT to more patients. The objective of this study is to create a prototype system which is capable of generating a patient's head and neck CTV contours from his CT scan. This paper summarizes our previous work [5-8] and presents a complete system with more comprehensive results.

### Imaging-based lymph node regions

The neck has an extensive lymphatic network [9]. In fact, more than one third of the body's total number of lymph nodes resides in the extracranial head and neck. Cervical lymph nodes are divided into regions or 'levels' that are described by their anatomic location [10]. Although this traditional classification was decided using surgical landmarks, translation into an imaging-based nodal classification is feasible.

Automatic segmentation of cervical lymph nodes remains to be an open problem, researchers [11,12] are actively working on techniques to segment the lymph nodes for diagnosis or surgery planning. However, in the context of radiation therapy planning, the exact contours of lymph nodes are not as important as the lymph node regions including the surrounding tissue which make up the CTV. Studies have been conducted to create an imaging-based classification for the lymph node levels of the neck that can be accepted by clinicians and easily used by radiologists [4,13-17]. Anatomic landmarks were chosen to create a consistent nodal classification similar to the clinically-based classifications. Radiologists must be able to identify the pertinent anatomic landmarks such as the bottom of the hyoid bone, the back edge of the submandibular gland, and the back edge of the sternocleidomastoid muscle. The Radiation Therapy Oncology Group (RTOG) [18] has also published guidelines for CT-based delineation of lymph node levels in the neck and the anatomic boundaries for delineation.

Automatic delineation of lymph node region can reduce physicians' manual CTV contouring time even though the results are not sufficiently accurate for clinical use directly [19,20]. Atlas-based segmentation is used in most of the state of the art research [17,21,22] and commercial tools [23,24] for automatic delineation of lymph node levels in head and neck CT. These methods tend to yield better results when the atlas is more anatomically similar with the target subjects. The method and database (CT images) used to construct an unbiased atlas is critical to the success of the segmentation [25,26]. However, the high anatomical variability in post-operative head and neck CT images makes it very difficult to construct a mean image and atlas that works well for all patients. We proposed an alternative approach which uses a collection of CT images with contoured CTV from previously treated patients as reference models [6], and a method to identify reference subjects whose anatomic structures share similar properties or features for a given target [8]. Using previously treated patients or canonical models with the most similar head and neck anatomy as references, an image registration process can segment lymph node regions more accurately for a target patient based on known contours in the reference models.

Recent studies evaluated some of the state of the art atlas-based segmentation tools listed above by comparing the automatically delineated head and neck lymph node region contours and volumes against the ones drawn by physicians [27-29]. In addition to qualitative assessment by physicians, statistics measures such as sensitivity and specificity or Dice similarity coefficient were commonly used as quantitative assessment. We also proposed an alternative quantitative evaluation using Hausdorff surface distance measure which maybe more clinically relevant than the statistical metrics [6].

Given the set of post-op head and neck cancer patients, a series of 2D contours were manually delineated for each of the lymph node levels on axial CT images; which build up to 3D volumes. Using an image registration technique, these expert drawn lymph node regions are used as reference models and templates to project the lymph node regions in another target image which are compared to the expert drawn contours in the target image, i.e. the "gold standard" or "ground truth". Instead of the atlas-based approach or choosing one patient as the reference model, we will determine criteria for choosing one or more similar reference models which can produce optimal results. Traditional 3D shape retrieval systems [30-32] mostly experiment with artificial models and focus mainly on classifying 3D models of very different shapes. While these experimental systems can match models of the same classes to a certain degree of success, they usually fall short of distinguishing the finer details of objects within a class. Using 3D medical images to find similarity among a known set of patients is becoming a research subject of interest in many medical domains. Ruiz *et al*. [33] use a shape-based similarity measure to find similar craniosynostosis patients for intervention planning. We developed a method to find similar head and neck cancer patients for radiation planning. The similarity of head and neck anatomy between patients is based not only on shape features of structures, such as outer body volume, mandible, and hyoid, but also on their relative locations. These types of medical-image-based problems are very domain specific, and are not solved by the traditional shape-based retrieval system.

Recent reviews of 3D shape matching techniques were done by Iyer [34] and Tangelder [35]. A majority of the 3D shape matching systems use feature-based methods, which compare geometric and topological properties of 3D shapes. Methods using features or distributions work reasonably well in classifying objects of different shapes, but they do not discriminate between objects of the same class such as the head and neck anatomy of different patients. The matching process is usually done by computing a distance between feature vectors representing the different objects. Most systems do not give many details on the distance measurements or their comparison methods, although they usually imply a Euclidian vector space model and use either a simple (weighted) Euclidean distance or a city-block (*L*_1 _Minkowski) distance.

## Methods

This prototype system is designed to take a cancer patient's head and neck CT images as input and use image registration techniques to produce projections of lymph node regions as output, which can be used to produce a CTV for the radiation treatment plan. The system can be divided into the following major components:

- Segmentation,

- Retrieval of similar reference models,

- Image registration.

**Figure 2:**
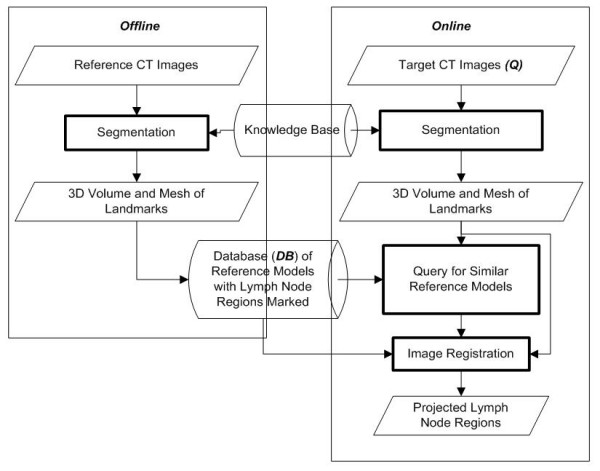
**System components block diagram**.

Figure 2 is the flow chart which shows how these components are linked. The offline process on the left creates a database **DB **of CT scans from prototypical reference patients on which experts have drawn contours that denote the lymph regions. These reference images {**d**_**i**_} are segmented offline to extract 3D volumes of landmark anatomical structures such as the mandible and hyoid [5]. 3D meshes and geometric properties of these 3D volumes are also stored in the database.

### Image registration

Given a reference model and a target patient's CT data set, we can use image registration methods to align the sets of CT images. Image registration is commonly used in medical imaging applications. It is essentially a process of finding a geometric transformation **g **between two sets of images, which maps a point **x **in one image-based coordinate system to **g(x) **in the other. By assuming the head and neck anatomy has similar characteristics between a specific target patient and a reference subject, we can use image registration methods to transform a region from the reference image set to the target image set.

Mattes and Haynor [36] implemented a multi-resolution non-rigid (deformable) image registration method using B-splines and mutual information. The transformation of a point **x **= [x, y, z]^T ^in the reference image coordinate system to the test image coordinate system is defined by a 3 × 3- homogeneous rotation matrix **R**, a 3-element transformation vector **T **and a deformation term **D**(**x|δ**):(1)

where **x**_*C *_is the center of the reference volume. A rigid body transformation defined by **R **and **T **was first calculated and used as the initial transformation for the deformation process. The deformation term **D**(**x|δ**) gives an x-, y-, and z- offset for each given **x**. Hence the transformation parameter vector μ becomes(2)

The first three parameters *γ*, *θ*, *ϕ *are the roll-pitch-yaw Euler angles of R. The translation vector **T **is defined by [*t*_*x*_, *t*_*y*_, *t*_*z*_]^T^. **T **and **R **together define the rigid body transformation.

The parameter **δ**_*j *_is the set of the deformation coefficients. The deformation was modeled on cubic B-splines [37] because of their computational efficiency (via separability in multidimensional expression), smoothness, and local control. The deformation is defined on a sparse, regular grid of control points **λ**_*j*_, each having associated x-, y-, and z-components of the deformation. The resolution(3)

of the deformation determines the spacing of the grid and can be anisotropic. Mattes uses control points on a uniform grid with spacing(4)

where *q*_*x*_, *q*_*y*_, and *q*_*z *_are the dimensions of the reference image.

The deformation at any point **x **= [*x*, *y*, *z*]^*T *^in the reference image is interpolated using a cubic B-spline convolution:(5)

By displacing the control points, intermediate deformation values are computed by cubic spline interpolation between them.

Because contract enhancement is often used in head and neck CT scans, image intensity range and distribution may vary in different data sets. A mutual information based registration method such as Mattes' can work with images in different range. However, the high anatomical variability in cancer patients' head and neck CT images particularly contributed by the surgical resections, pure voxel intensity based registration such as Mattes' method described in previous section does not always produce satisfactory results. A new method [6] is developed which integrates landmark based information with intensity scheme; hence CT data set need to be preprocessed to extract landmark information.

### Segmentation and landmark correspondence

Fully automatic segmentation in the neck region is particularly difficult, because many soft tissue anatomic entities are small in size and similar in density. Furthermore, they can be directly adjacent to each other or only divided by fascial layers that are not visible in CT images. The relative locations between anatomic entities can vary in different axial locations. Little work has been done specifically for the neck images; few exceptions include the work of Krugar *et al*. [10] who implemented a semi-automatic system to segment neck CT images for pre-operative planning of neck dissections; and the work of Cordes *et al*. [38] who developed NeckVision system for neck dissection planning. We implemented an automatic segmentation method [5] designed to locate anatomic structures in the neck that are relevant to the lymph node region boundaries including cervical spine, mandible, hyoid, jugular veins, and carotid arteries. This method is motivated by a knowledge-based technique [39] using consists of constraint-based dynamic thresholding, negative shape constraints to rule out infeasible segmentation, and progressive landmarking that takes advantage of the different degrees of certainty of successful identification of each structure.

The drawback of this 2D thresholding approach is the difficulty in determining the optimal threshold, especially in the head and neck region where many structures of similar density are crowded in a tight space. Our proposed method eliminates the need of finding the optimal threshold by combining the 2D thresholding with a 3D active contour procedure (by ITK, www.itk.org). A sub-optimal threshold that only produces partial structure on some axial slices can grow into a 3D volume through 3D active contouring. The 2D regions produced by the knowledge-based dynamic thresholding method can be used as the initial region or "seed" to eliminate the need of user input. In the context of finding relevant landmarks, it is not necessary to fully segment certain structures such as the blood vessels and their branches as we are only interested in sections lateral to the hyoid. This lax requirement circumvents the need of perfect segmentation and optimal active contouring parameters. These selected structures usually have clear contour allowing successful segmentation within the section of axial slices of interests.

**Figure 3:**
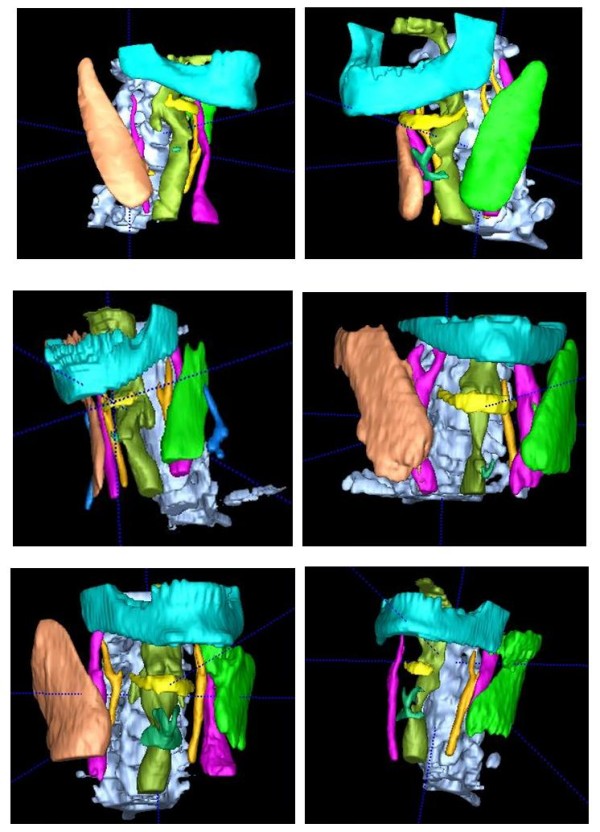
**Examples of automatic segmentation results for selected subjects: (a) cervical spine, (b) respiratory tract, (c) mandible, (d) hyoid, (e) thyroid cartilage, (f) jugular vein, (g) common carotid artery, and (h) sternocleidomastoid muscle**.

The anatomical structures of interests are segmented in the order according to the reliability of successful detection. In addition to domain knowledge of gray tone range, size and shape, each structure is also associated with location relative to other structures that can be found prior to itself. For each structure, we first run the dynamic thresholding process to find 2D regions in axial slices according to domain knowledge. Note that some axial slices may yield successful 2D segmentation results and others do not, we then the 3D active contouring to build a 3D model using the partial 2D segmentation result as the initial seed. Also note that the active contouring may not grow the structure perfectly to its full extent, but sufficient to provide landmark information. This two step process is then repeated for the next structure. 3D surface meshes of those structures are constructed for both the reference and target subjects. The meshes can be used as landmarks to improve the alignment and to measure similarity between the subjects. Figure 3 shows examples of segmentation results on selected subjects.

We choose to use these 3D anatomic structure surfaces as landmarks because virtually no 1D (points) or 2D (lines) anatomic features that can be used as landmarks are defined in the neck. By using Shelton's method [40] of finding surface mesh correspondence, we can estimate correspondence between the surfaces of the landmark anatomic structures of the reference and target subjects. The following energy function for which smaller values indicate better correspondences is defined to evaluate possible correspondence relations:(6)

where **C **is the function that maps points on surface **A **to matching points on surface **B**, *α *and *β *are weight parameters, ***E***_sim _is the similarity term which measures how closely **C **matches points on **A **to points on **B**, ***E***_str _is the structural term that minimize the distortion of surface **A**, and ***E***_pri _is the "prior information" term which ensures **C **represent a plausible deformation. Initial values of *α *and *β *are set to 0.001 and 0.0001 respectively.

Let ***υ***_*k *_be the set of landmark points sampled from the mandible, hyoid and other surface meshes of the reference image set or surface **A**, and **θ**_k _be their corresponding locations on surface ***C***(**A**) or the transformed surface which matches the test image set and minimize the energy function. The deformation **ζ **at those landmark points is simply(7)

### Image registration with landmark correspondence

Given the anatomic structure surface correspondence between the reference and target data, we developed a method to incorporate these landmarks into the image registration process. Instead of initializing the deformations at the control points to zeros or random numbers as in the Mattes method, we can use the landmark correspondence to initialize or adjust the deformations at the control points at each of the multi-resolution stages. The deformation control points are set to a uniform grid(8)

where 0 ≤ *l *≤ *ρ*_*x*_, 0 ≤ *m *≤ *ρ*_*y*_, 0 ≤ *n *≤ *ρ*_*z*_, and the corresponding deformation values **D**(**λ**_*j*_) are either initially set to zero or calculated from the deformation coefficients **δ**_*j *_of the previous iteration at a lower resolution of control points as in equation (5).

Given **ν**_*k *_and **ϖ**_*k *_as sets of corresponding landmarks in the reference and target images described in equation (7), the deformation of each control point that has landmark points in close proximity is modified to the deformation of the closest landmark point as follows(9)

where **υ**_*k *_is the closest landmark point to **λ**_*j *_in the reference image set, and **ζ**_*k *_is the deformation at **υ**_*k *_obtained from the surface correspondence in equation (7). A new set of deformation coefficients **δ **is then set to the spline coefficients of the new grid of deformation values **D'**(**λ**). While the new **D' **might not be initially smooth, the following mutual information registration will mitigate the side effect. Finally the transformation parameter vector **μ **is input to the optimizer for alignment.

### Similarity measurement based on anatomical structure geometry

As we need to compare similarity between anatomic structures from different patients, we do so by measuring the errors between structure surfaces using the 3D Hausdorff distance [41]. Given two surface meshes, *S*_*R *_and *S*_*T*_, the distance between a point *p*_*R *_belonging to *S*_*R *_and the mesh *S*_*T *_can be defined as follows:(10)

We first align meshes *S*_*R *_and *S*_*T *_with the Iterative Closest Point (ICP) rigid body registration [42] so they are roughly in the same 3D coordinate. Given the 3D point sets *P*_*R *_= {*p*_*i*_} containing the n vertices of *S*_*R*_, the registration process will produce a transformation matrix ***T ***which minimizes the function(11)

The transformed reference mesh ***T****S*_*R *_consists of vertices {***T***_*pi*_}, and the Hausdorff distance between ***T****S*_*R *_and *S*_*T *_is given by(12)

A database ***DB ***of CT scans is created from prototypical reference subjects on which experts have drawn "ground truth" contours that denote the lymph node regions. These reference data sets {***d***_*i*_} are segmented offline to extract 3D volumes of identifiable structures, including the mandible, hyoid, jugular veins and the outer body contour that are relevant to the boundaries for the lymph node regions [5]. We use these landmark anatomic structures to rapidly produce a distance metric between a target CT scan as query ***Q ***and each data set ***d ***in ***DB***. The feature vectors that we use to compare two CT scans include three kinds of features: 1) simple numeric 3D regional properties of these structures, such as volume and extents, 2) vector properties or the relative location between structures and 3) shape properties or the surface meshes of these structures. The feature vector consists of the following properties,

- volume and extents of the overall head and neck region,

- surface meshes of the mandible and outer body contour,

- 3D centroid difference vector between mandible and hyoid,

- 2D centroid difference vectors between hyoid and jugular veins, and between hyoid and spinal cord on the axial slice at the centroid of the hyoid,

- normalized centroid locations of the hyoid and the mandible within the region.

Given feature vectors *F*_*d *_and *F*_*Q *_for model ***d ***and query ***Q ***in the feature vector space R^N^, the following weighed Euclidean distance is used as the distance measure:(13)

where *w*_*i *_is the weight parameter, *d*_*i *_is the distance function for feature *i*,(14)

*d*_*h *_is the Hausdorff distance defined in equation (12), and ***T ***is the ICP registration transformation matrix. The weight parameters range from 10 to 0.1 from heavy to light in the order of: 1) hyoid locations (normalized and relative to other structures), 2) mandible distance (between two models), 3) vertical distances between skull base, mandible and, hyoid, 4) head and neck outer contour and volume, and so on.

**Figure 4:**
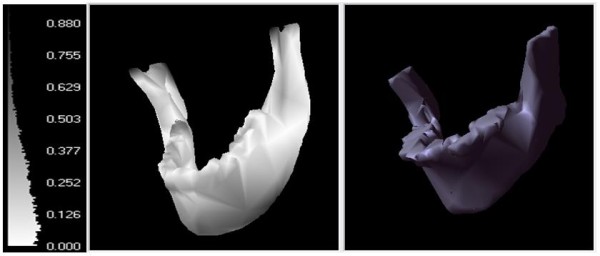
**Measuring distance *d*(*p*_*R*_, *S*_*T*_) in between sample point *p*_*R *_on reference mesh surface on the left and target mesh *S*_*T *_on the right**. The bar graph on the left indicates distance measurement in centimeter corresponding to the shade of *S*_*R*_.

The distance between mandible meshes of two subjects is one major discriminating feature of the proposed distance measure. Figure 4 shows the measurement of point to surface distance *d *as in Equation (10), from which the directional Hausdorff distance *d_h _*between the reference mandible surface mesh to the target mesh is derived. The mesh on the left with shading indicates the distance from a given point on the surface to the mesh on the right.

## Results and discussion

CT images are acquired from the radiation therapy treatment planning system. Head and neck cancer patients who did not have large scale surgical resection are the primary candidates. All the images used in the experiments are CT scans performed at the University of Washington Medical Center using a General Electric CT scanner. Head and neck CT images were selected in which all of the slices are 512 × 512 pixels in axial dimension; the distance between slices varies between 1.25 mm and 3.75 mm for each image set. Selected lymph node regions are drawn as 2D contours on axial CT slices of twenty subjects chosen by resident physicians in University of Washington Radiation Oncology Department. These lymph node regions are used as "ground truth" or "gold standard" as we evaluate the computer generated lymph node regions. A simple automated segmentation pre-process removes the bed and immobilization devices on each CT images. The experiment was conducted on personal computers with Pentium 4 processors and 2 GB RAM.

### Image registration

We ran the image registration experiment with the twenty sets of CT images chosen by the oncologists, each of which is used as a reference and a target data set to align with every other image set. The maximum resolution of the deformation control points as defined in Eq. (3) is [15,15,11], or 2475 control points. The pairing results in 20 × (20-1) = 380 total registration tests. We used the results of the Mattes method as the baseline to compare with the results of the new method.

Out of the 380 total tests, the Mattes method failed to converge in 13 cases while the proposed method succeeded in all cases. The average time to complete the registration improved by approximately 20%. Tables 1 and 2 compares the success rate and convergence time of the two image registration method out of 380 inter-subject cases.

**Table 1:** Success rate comparison between Mattes and proposed method.

	Total cases	Successful registration cases	Success rate (%)
Mattes method	380	367	96.57%

Proposed method using landmark correspondence	380	380	100.00%

**Table 2:** Time of convergence comparison between Mattes and proposed method.

	Average time of convergence	Standard deviation time of convergence	Cases faster
Mattes method	32 minutes	6 minutes	326/380

Proposed method using landmark correspondence	26 minutes	5 minutes	54/380

3D volumes and surface meshes of the lymph node regions are built from the 2D "ground truth" contours as well as the computer generated lymph node regions. We can quantitatively evaluate the results of the image registration process with statistical metric such as spatial overlap index and surface distance measure. Given *V*_*T *_as the ground true volume and *V*_*C *_as the computer generated volume, we can use Dice similarity coefficient (DSC) to measure the spatial overlap between two segmentations [43], where(15)

Table 3 shows examples of the statistical evaluation of the automatically delineated contours. While statistical metrics give certain validation, we suggest that Hausdorff surface distance error may be more clinically relevant as it represents the "worst case scenario" or often a critical error. Given two 3D surface meshes: reference lymph node region transformed into target space as the projected region and the corresponding "ground truth" region of the target subject; we measure the Hausdorff distance and mean distance between these meshes by sampling the surface points. The Hausdorff distance between the projected lymph node region *N *of reference subject *S*_*R *_and the expert drawn region for a given target subject *S*_*T *_can be defined as *D*_*H*_(***T****S*_*R*_, *S*_*T*_, *N*), where ***T ***is transformation resulted from the image registration of *S*_*R *_and *S*_*T*_.

**Table 3:** Example of volume overlap analysis with Dice similarity index (DSC).

Level	Side	Average DSC	Standard deviation
1B	Left	0.74	0.11

1B	Right	0.76	0.10

2	Left	0.71	0.12

2	Right	0.72	0.12

**Figure 5:**
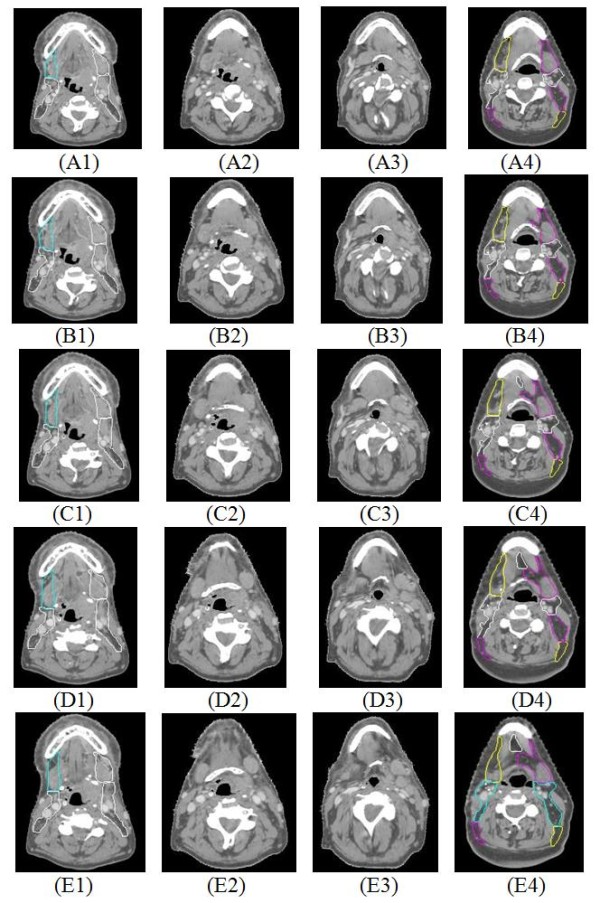
**Comparison of results from the image registration methods with and without using landmark correspondence**. Rows A-E show selected axial CT slices in the neighborhood of the hyoid in various data sets from superior to inferior. Column 1 shows slices from the reference subject, column 4 from the target subject, column 2 is the result of Mattes' image registration method, and column 3 is the result of the new method using landmark correspondence.

Figure 5 shows an example of qualitative comparison of image registration results using the Mattes' method without landmark information and the proposed method which incorporates landmark correspondence. Rows A-E show selected axial CT slices in the neighborhood of the hyoid in various data sets from superior to inferior location. Column 1 shows slices from the reference subject, and column 4 shows slices from the target subject. Columns 2 and 3 show transformed reference images which were re-sampled to match or align with the target images using the transformation function **g**(**x|***μ*) of equation (1) produced from the image registration procedure. Column 2 is the result of Mattes' image registration method without using landmark information, and column 3 is the result of the proposed method using landmark correspondence. The images in column 3 match better qualitatively to column 4 in several ways. First of all, for example, the outer body contours is closer to the ones of target CT. Second, the superior-inferior location of the hyoid bone and the mandible in column 3 match more closely to the ones in column 4; such as the hyoid bone is in slices (rows) C-E (and beyond) of column 2, but in slices A¬D of column 4, and the inferior boundary of the mandible ends in slice D of column 2, but continues beyond slice E in columns 3 and 4. This is important because both hyoid and mandible represent superior-inferior boundaries to adjacent lymph node levels. Third, the proximity of the cervical spine in column 3 appears to be more closely matched to column 4. Even though certain soft tissue structures appear distorted in column 3; in the context of delineating lymph node regions, it is relatively less important compared to matching the location of the dominant structures defining the boundaries.

**Figure 6:**
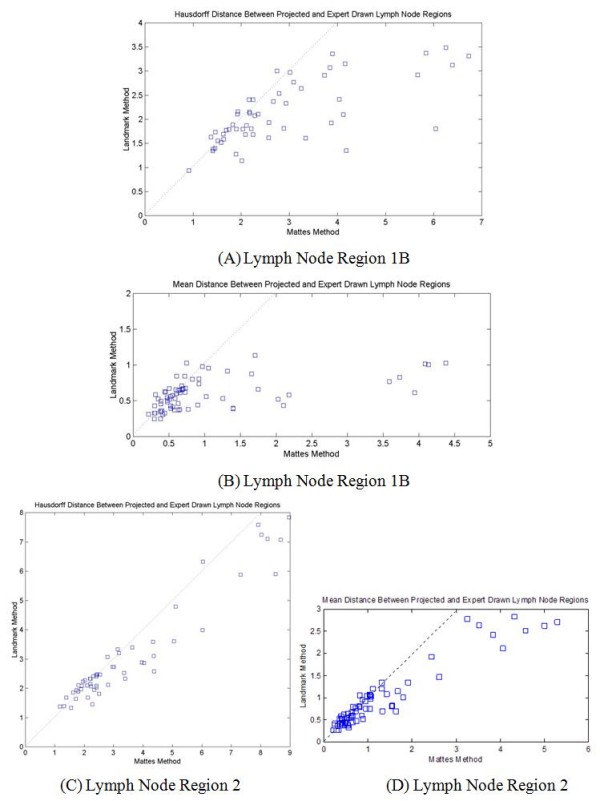
**Hausdorff and mean distance (in cm) between transformed reference mesh and the target mesh of nodal regions for all *S*_*R *_and *S*_*T*_, comparing image registration results from Mattes method and the proposed landmark method**.

Figure 6 shows comparisons of image registration results from the Mattes method and the proposed method incorporating the landmark correspondence. The horizontal axis represents the Hausdorff or mean distance between projected lymph node regions based on the reference model using image registration results from the Mattes method and corresponding expert drawn lymph node regions of the target subject. The vertical axis represents matching results using the proposed registration method. The diagonal dotted lines represent the points where the two measures are equal. The figures show overall improvement using the proposed registration method. Tables 4 and 5 compare the mean and standard deviation of results from two methods. The new landmark-based method improved the average Hausdorff distance by as much as 25%, and the average mean distance by as much as 42%.

**Table 4:** *D*_*H*_(*TS*_*R*_, *S*_*T*_, *L*) for all *S*_*R*_, *S*_*T*_. Hausdorff distance (in cm) between transformed reference mesh and target mesh for nodal levels (*L*) 1B and 2.

Level	Algorithm	Average	Standard deviation
1B	Mattes method	2.85	1.44

1B	Landmark enhanced	2.12	0.64

2	Mattes method	3.48	2.15

2	Landmark enhanced	3.07	1.75

**Table 5:** Mean distance (in cm) between transformed reference mesh and target mesh for nodal level 1B and 2.

Level	Algorithm	Average	Standard deviation
1B	Mattes method	1.02	1.01

1B	Landmark enhanced	0.59	0.21

2	Mattes method	1.21	1.20

2	Landmark enhanced	0.91	0.68

There is currently no quantitative standard defining clinical acceptability for automatically delineated target contours, whether in statistical error or maximum surface distance error. However, as the results for two sample lymph node region shown in Tables 4 and 5, the average distances (error) between the generated CTV and ground truth are under 1 cm for the cases tested; the Hausdorff distances are still in the 3 cm range. Methods are being investigated to reduce the maximum error as part of the future work for this continuing project. One of which is to port the registration module to a supercomputer platform and increase to resolution of the control points during the deformation process. Another is to merge the results of projected CTV from multiple similar reference models and possibly alleviate regions that contribute the worst Hausdorff distance.

**Figure 7:**
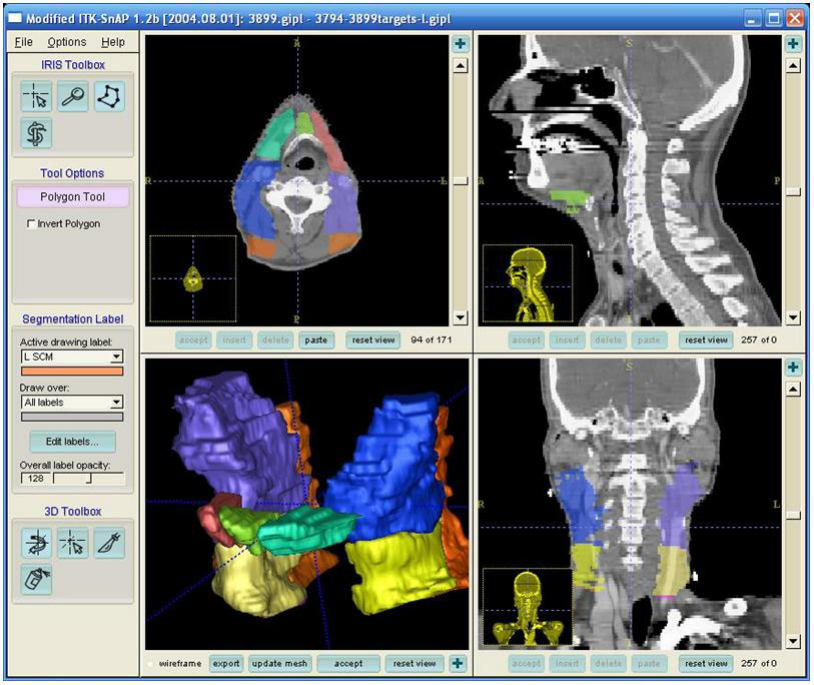
**Sample result of lymph node region projection: (a) level IA, (b)(c) level IB, (d)(e) level II, (f)(g) level III, and (h)(i) level V. Each color region corresponds to a lymph node region**.

Figure 7 shows an example of the projected lymph node region contour based on the result of the image registration. The projected 3D lymph node regions are shown at the lower left 3D view. They are also overlaid on the target CT images in axial, sagittal, and coronal views. The radiation oncologists can make adjustments to the suggested contours as needed.

While the proposed method improves the overall results, it does not decrease the Hausdorff or mean distance in every case. One reason is that some of the "ground truth" contours deviate from the image-based classification because physicians were sometimes influenced by their clinical judgment as they outline the contours [20]. We also observed that for cases which already produce small Hausdorff or mean distance, the improvement or regression tends to be negligible. As the collection of reference data sets grow, it becomes critically important to locate the candidates which can potentially produce the best results based on the similarity measurement.

### Similarity measurement

For the twenty selected subjects with expert drawn "ground truth" lymph node regions, each of which is used as target and reference, we measure the feature-vector-space distance between each target-reference pairs. For each target subjects, we rank all of the reference subjects by two separate measurements:

- Ranking *R*_*I *_ranks by Hausdorff distance *D*_*H *_between projected lymph node region and corresponding expert drawn region of target subject. The smallest value has the highest rank. This represents the ranking of similarity according to the image registration results, assuming that the more similar reference image aligns better with the target image. Note that there may be different rankings for each lymph node region as the Hausdorff distance results vary. These rankings are used as the baseline.

- Ranking *R*_*F *_ranks by distance measure in the feature vector space *D*_*F *_in Equation (13). The smallest value has the highest rank. This represents the ranking of similarity according to the geometrical features.

To validate the similarity ranking based on geometrical features *R*_*F*_, we compare it to the ranking based on image registration results for each target subject *R*_*I*_. We define *R*(*i*, *S*_*T*_) as the *i*th reference subject in the ranking for the target subject *S*_*T*_. For 81% of the instances, *R*_*F*_(1, *S*_*T*_) is the same as *R*_*I*_(1, *S*_*T*_), meaning that 81% of the most similar reference subjects according to geometrical features matches the one that align best with the target according to image registration. In other words, the probability P(*R*_*I*_(1, *S*_*T*_) = *R*_*F*_(1, *S*_*T*_) | *R*_*F*_(1, *S*_*T*_)) = 0.81. Additionally, *R*_*F*_(1, *S*_*T*_) has 96% chance of matching one of the top three subjects in *R*_*I*_. Table 6 summarizes the probabilities of the most similar subject based on geometric features matching subjects that align best with the target.

**Table 6:** Probabilities of the most similar subject based on geometric features matching subjects that align best with target.

	*x *= 1	*x *= 2	*x *= 3	*x *> 3
P(*R*_*I*_(*x*, *S*_*T*_) = *R*_*F*_(1, *S*_*T*_) | *R*_*F*_(1, *S*_*T*_)), where *R*_*F*_(1, *S*_*T*_) is the most geometrically similar reference subject	81%	11%	4%	4%

As we previously defined *D*_*H*_(*TS*_*R*_, *S*_*T*_, *n*) as the Hausdorff distance between the projected lymph node region *n *based reference subject *S_R _*and the expert drawn for a given target subject *S*_*T*_. The Hausdorff distance based on the most similar reference subject in the feature space *R*_*F*_(1, *S*_*T*_) becomes *D*_*H*_(*TR*_*F*_(1, *S*_*T*_), *S*_*T*_, *n*). Table 7 compares the image registration results of the most similar reference subject to results of all 380 test cases listed in previous section. The average *D*_*H *_of 1.28 cm is approximately 50% improvement over the average of all test cases.

**Table 7:** Compare image registration results based on most similar reference subject and results based on all test cases.

	Average	Standard deviation
*D*_*H*_(*TR*_*F*_(1, *S*_*T*_), *S*_*T*_, *n*) for all *S*_*T *_and *n*, based on most similar reference subjects of each target subject.	1.28	0.31

*D*_*H*_(*TS*_*R*_, *S*_*T*_, *n*) for all *S*_*R*_, *S*_*T *_and *n*, based on all combinations of reference and target subjects.	2.59	0.90

**Figure 8:**
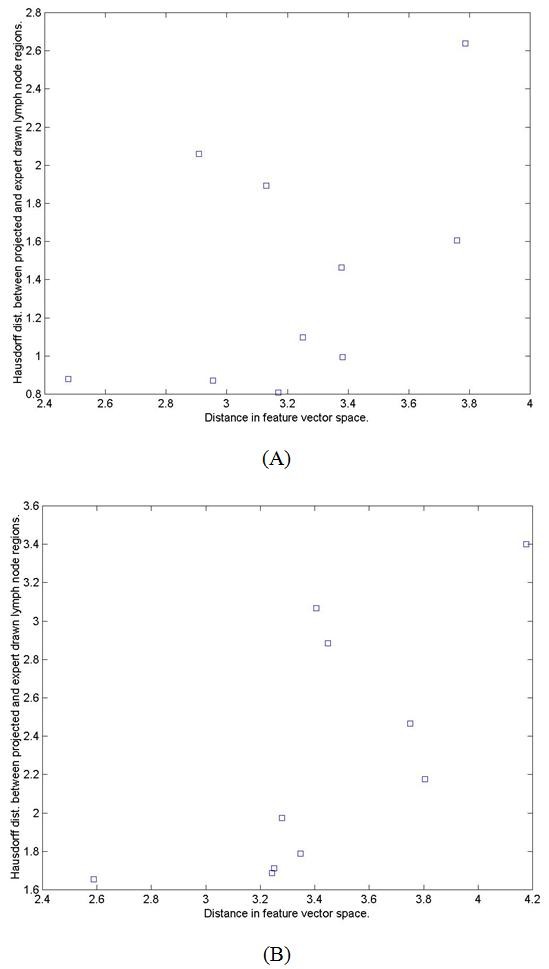
**Examples of correlation between the proposed distance measure *D*_*F *_in feature vector space (horizontal axis) and the Hausdorff distance *D*_*H *_between the projected lymph node regions resulting from registration and those hand-drawn by experts (vertical axis)**. Figures A and B compare correlation for two different lymph node regions. Each point in the figures correspond a test subject.

Figure 8 shows examples of correlation between the proposed distance measure and the result of 3D deformable image registration for selected target patients. The vertical axis shows the Hausdorff distance *D*_*H *_between the transformed 3D mesh of the reference model lymph nodal region and the corresponding mesh of the test model using the transformation produced by the 3D deformable image registration. The horizontal axis represents the distance measurement, or the weighed Euclidian distance *D*_*F *_between the test and reference model properties in the feature vector space. The leftmost point in each graph represent the most similar reference subjects *R*_*F*_(1, *S*_*T*_) which matches to one of the subjects that align well with the target, i.e. with the shortest Hausdorff distance *D*_*H*_(*TR*_*F*_(1, *S*_*T*_), *S*_*T*_, *n*) compares to others. Table 8 also shows statistical analysis of the correlation coefficients of *D*_*F *_and *D*_*H *_for all the target subjects *S*_*T*_.

**Table 8:** Correlation coefficients of *D*_*F *_and *D*_*H *_for all the target subjects *S*_*T*_.

	Average	Standard deviation
Correlation_coefficient(*D*_*H*_, *D*_*F*_) for all *S*_*T*_.	0.61	0.19

### Qualitative evaluation

**Figure 9:**
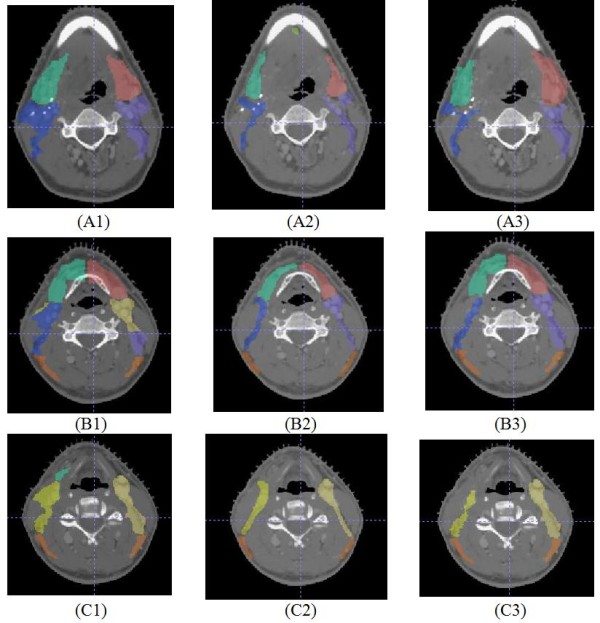
**Comparison between projected lymph node regions and expert drawn regions**. Column 1 on the left shows projected regions from Mattes' method; column 3 on the right shows results from the new method using landmark information. Regions in column 2 are drawn by a radiation oncologist and considered to be clinically acceptable.

The projected lymph node regions can be evaluated qualitatively by superimposed on the target subject's CT images. Figure 9 compares projected lymph node regions to expert drawn regions, each region is color coded. Rows 1-3 are sample CT slices from superior to inferior positions of the same target subject. Column 1 on the left shows projected regions from Mattes' method; column 3 on the right shows results from the new method using landmark information. Regions in column 2 are drawn by a radiation oncologist. These projected lymph regions are reviewed by the radiation oncologist and considered to be clinically acceptable. The results from the Mattes' method are more generous in certain areas covering muscle tissues. Although it may be considered harmless today, it can be less desirable as the precise lymph node region contours becomes more important in the future.

## Conclusion

This software system is the first of its kind that attempts to automate the process of identifying Clinical Target Volume for head and neck cancer radiation treatment by projecting lymph node regions using inter-subject image registration techniques. We developed a image registration technique using landmark correspondences in conjunction with a voxel based mutual information method, along with a patient similarity measurement using the 3D geometrical relationship between anatomic structures in addition to 3D shape descriptors of the structures. The image registration technique provides a way to potentially automate the lymph node region contouring process for radiation therapy planning. While the alignment of the projected lymph node contours on the target image are close enough to suggest the technique has promise, the results do not conform to clinical criteria. However, this system could drastically reduce the time needed for a physician to draw a fully-detailed target volume as an intermediate step that suggests an initial target volume upon which the physician can adjust and refine before it is used for therapy.

While the new image registration technique improves the overall result of the target volume projection, the results discussed in previous section shows that it is perhaps more important to identify the reference models which can potentially produce the best alignment. As the similarity measurement is validated by the target volume projection, twenty data sets with "ground truth" information is not quite enough to provide a variety of subjects with different anatomic features. We will expand the test data set to refine and improve the method, while continuing to investigate other potential features that can be incorporated in the similarity measure for better results, including more anatomic structures and their relationships. In addition to using one reference model to project the CTV for a test subject as in the current method, we will investigate the possibility of using multiple similar reference models and merge their CTV projects to further reduce the maximum error.

These software tools will need to be evaluated at a clinical environment for head and neck cancer patients who undergo 3D conformal radiation therapy where the computer generated target contours can be compared to the expert drawn contours of more target subjects. The method can also be generalized for treatment of other types of cancer. Furthermore, this work can potentially be integrated with other research work such as predictions of the regional lymphatic involvement of head and neck squamous cell carcinoma based on primary tumor location and T-stage [44]. By using the lymphatic prediction, we can delineate proper target volumes for given primary tumor location and T-stage.

## Competing interests

The authors declare that they have no competing interests.

## Authors' contributions

CT carried out most of the design and implementation. LS participated in its design. IK conceived of the study, and participated in its design and coordination with the clinicians. All authors read and approved the final manuscript.
